# Ataxia‐telangiectasia mutated activation mediates transforming growth factor beta signaling in acetaminophen‐induced liver injury in mice

**DOI:** 10.14814/phy2.70695

**Published:** 2025-12-09

**Authors:** Matthew McMillin, Christopher S. Chu, Elaina Williams, Juliet Venter, Kiersten Bell, Anca D. Petrescu, Patrick Mireles, Sharon DeMorrow

**Affiliations:** ^1^ Huffington Department of Education, Innovation & Technology Baylor College of Medicine Temple Texas USA; ^2^ Department of Medicine Baylor College of Medicine Temple Texas USA; ^3^ Division of Pharmacology and Toxicology, College of Pharmacy The University of Texas at Austin Austin Texas USA; ^4^ Department of Internal Medicine Dell Medical School, The University of Texas at Austin Austin Texas USA

**Keywords:** acute liver injury, DNA damage, DNA damage response, KU55933, SMAD signaling

## Abstract

Acetaminophen (APAP) overdose is associated with increased transforming growth factor beta 1 (TGFβ1) signaling and elevated oxidative stress, which exacerbate DNA damage. TGFβ1 has been shown to regulate ataxia‐telangiectasia mutated (ATM) signaling and DNA repair in other cell types. This study investigates the DNA damage response (DDR) during APAP‐induced liver injury, focusing on ATM‐mediated regulation of TGFβ1 signaling. APAP administration in vitro and in vivo resulted in DNA damage, increased ATM signaling, accumulation of γH2AX, and activation of phosphorylated ataxia telangiectasia mutated (pATM) and phosphorylated checkpoint kinase 2 (pChk2). Pretreatment with an ATM inhibitor, KU55933, attenuated APAP‐induced hepatocyte damage and resulted in attenuated mothers against decapentaplegic homolog 2/3 (SMAD2/3) signaling with no changes in activated TGFβ1 levels, suggesting that ATM activation modulates TGFβ1 signaling via post‐translational mechanisms. APAP was found to promote transforming growth factor beta receptor 2 (TGFβRII) stabilization through activation of phosphorylated casitas B‐lineage lymphoma (p‐c‐cbl) and subsequent neddylation of TGFβRII, which was attenuated by inhibitors of ATM signaling or neddylation machinery. In conclusion, APAP‐induced hepatic DNA damage activates an ATM‐mediated response that enhances TGFβ1 signaling through stabilization of TGFβRII, and inhibition of ATM consequently reduces APAP‐induced hepatic injury.

## 1 INTRODUCTION

Acetaminophen (APAP) is a common analgesic/antipyretic that is widely used throughout the world, and over 20% of the United States population ingests APAP weekly (Kaufman et al., 2002). APAP is safe at therapeutic dosages but induces acute liver injury when the therapeutic window is exceeded. Drug‐induced liver toxicity accounts for 50% of the cases of liver failure, with APAP being the leading cause of acute liver failure in Western societies (Lee, 2013). Acute liver failure is associated with increased levels of inflammation, oxidative stress, and hepatic cell death. At this time, treatment methods are limited outside the therapeutic window for the APAP toxicity antidote *N*‐Acetylcysteine (Bernal et al., 2010).

APAP intoxication leads to the accumulation of the highly reactive metabolite N‐acetyl‐*p*‐benzoquinone imine (NAPQI) (Dahlin et al., 1984; Manyike et al., 2000). NAPQI is normally neutralized via conjugation with glutathione (GSH), subsequent conversion to cysteine or mercapturic conjugates, and is excreted in urine and bile (Ghanem et al., 2016; Potter & Hinson, 1987). However, during APAP intoxication, NAPQI accumulates and GSH is depleted, resulting in reduced hepatic antioxidant capacity (Jollow et al., 1973). The lowered antioxidant capacity, coupled with increased reactive metabolites, induces oxidative stress, which results in DNA damage (Borude et al., 2018; Dizdaroglu, 2012; Ghanem et al., 2016). In addition to direct damage to DNA bases, oxidative stress can disrupt the DNA backbone, causing single stranded breaks (SSBs), and if not repaired, further results in double strand breaks (DSBs), a catastrophic form of DNA damage.

To prevent cellular injury, cells have developed mechanisms to sense DNA damage and elicit a DNA damage response (DDR) (Chou et al., 2015; Cortez et al., 2001; Rasmussen & Painter, 1964). DSB repair is initiated by the recruitment of the MRN complex (Mre11, Rad50, and Nbs1) to the site of the DSB, which in turn recruits ataxia telangiectasia mutated (ATM) to phosphorylate histone H2AX on Ser139 (γH2AX) (Rogakou et al., 1998; Stracker et al., 2013; Sun et al., 2005). Phosphorylation at this site provides a docking site for tumor protein p53‐binding protein 1 (53BP1), allowing ATM to directly increase phosphorylated checkpoint kinase 2 (pChk2), thereby regulating the transcription factors responsible for controlling cell cycle arrest, cellular senescence, and/or apoptosis (Chatterjee & Walker, 2017; Matsuoka et al., 2007; Shiloh & Ziv, 2013). Previous studies have shown that nuclear DNA fragmentation occurs during APAP‐induced toxicity in mice and humans (McGill et al., 2012; Shen et al., 1992). Likewise, DNA repair pathways are activated in mice exposed to a dose of 300 mg/kg APAP while they are inactivated after a dose of 600 mg/kg APAP (Borude et al., 2018). In vitro, APAP has been shown to activate ATM signaling and cell cycle arrest (Bandi et al., 2014; Gupta et al., 2020; Viswanathan et al., 2018, 2021), suggesting that ATM signaling could play a role in APAP toxicity, though this has not been definitively demonstrated to date.

Transforming growth factor beta 1 (TGFβ1) is a cytokine that plays a role in numerous cellular processes. TGFβ1 signal transduction occurs through the binding of a heterotetramer receptor complex consisting of TGFβ receptor I (TGFβRI) and TGFβ receptor II (TGFβRII) and the subsequent phosphorylation of mothers against decapentaplegic homolog 2 (SMAD2) and mothers against decapentaplegic homolog 3 (SMAD3) (Abdollah et al., 1997; Zhang et al., 1996). Considerable crosstalk between ATM signaling and TGFβ signaling has been shown in response to DNA damage (Wang et al., 2013; Wiegman et al., 2007). Specifically, ATM activation increases phosphorylated casitas B‐lineage lymphoma (p‐c‐cbl), which promotes the covalent conjugation of neuronal precursor cell‐expressed developmentally downregulated protein 8 (NEDD8) to TGFβRII (Li et al., 2019). This process is called neddylation and prevents TGFβRII ubiquitination‐dependent degradation. Multiple studies have shown that an upregulation of TGFβ1 occurred during acute liver injury and is a driving factor for necrosis and senescence (Bird et al., 2018; McMillin et al., 2014, 2019). Additionally, patients with APAP overdose have increased hepatic TGFβ mRNA and increased plasma TGFβ1 concentrations (Miwa et al., 1997), indicating that TGFβ1 plays a role in the progression of acute liver failure.

There is a current gap of knowledge regarding the relationship between ATM signaling and TGFβ1 in response to APAP toxicity. The aims of this study are to: (i) investigate the ATM signaling in APAP‐induced liver damage in vivo using a mouse model of APAP toxicity; (ii) validate the involvement of ATM signaling in APAP‐caused injury in hepatocytes in vitro using a non‐cancerous cell line; (iii) demonstrate the role of ATM in APAP‐induced TGFβ1 signaling. This work is based on a Ph.D. dissertation of Dr. Christopher Chu (Chu, 2023).

## 2 MATERIALS AND METHODS

### 2.1 Reagents and materials

Acetaminophen (Cat# A7085) was from Sigma‐Aldrich (St Louis, MO). Thaizolyl blue dye (MTT) (Cat# M2128) was purchased from Sigma‐Aldrich (St Louis, MO). KU55933 (Cat# 3544) and MLN4924 (Cat# 64‐991‐0) were procured from Tocris (Minneapolis, MN). Dimethyl sulfoxide (Cat# 276855) was from Millipore Sigma (St Louis, MO). Cell lysis buffer was comprised of 0.1 M TRIS, 5 M NaCl, 0.5 M EDTA, IGEPAL. Antibodies were procured from ThermoFisher (Waltham, MA) for pChk2 (Cat# PA5104715). Bovine serum albumin (BSA) (Cat# BP9706) was purchased from ThermoFisher (Waltham, MA). Antibodies were acquired from Cell Signaling (Danvers, MA) for phospho‐ATM (Cat# 13050), ATM (Cat# 2873), phospho‐H2AX (Cat# 9718), phospho‐SMAD2 (Cat# 3108), phospho‐SMAD3 (Cat# 9520), TGFβ Receptor II (Cat# 79424), p‐c‐cbl (Cat# 8869), apoptosis‐inducing factor (AIF; Cat# 4642), and NEDD8 (Cat# 27455). Antibodies were purchased from Abcam (Cambridge, United Kingdom) for TGFβ1 (Cat# ab92486), SMAD2 (Cat# ab40855), SMAD3 (Cat# ab40854), and cytochrome P450 2E1 (CYP2E1) (Cat# ab28146). Antibodies were procured from GeneTex (Irvine, CA) for GAPDH (Cat# GTX627408). C‐Jun N‐terminal Kinase (JNK) (Cat# SC‐7345) and phosphorylated c‐Jun N‐terminal kinase (pJNK) (Cat# SC‐6254) antibodies were procured from Santa Cruz Biotechnology (Dallas, TX). Secondary antibodies for immunofluorescence were purchased from Jackson ImmunoResearch Laboratories (West Grove, PA) for AlexaFluor 488 anti‐rabbit (Cat# 711‐545‐152), AlexaFluor 488 anti‐mouse (Cat# 711‐545‐150), Cy3 anti‐rabbit (Cat# 711‐165‐152), and Cy3 anti‐rabbit (Cat# 711‐165‐150). Vectastain Elite ABC HRP kits were bought from Vector Laboratories (Burlingame, CA) for anti‐mouse (Cat# PK‐6102) and anti‐rabbit (Cat# PK‐6101). Western blot secondaries were purchased from Leica Biosystems (Wetzlar, Germany) for anti‐mouse IR‐Dye 680RD (Cat# NC0250903) and anti‐rabbit IR‐Dye 800CW (Cat# NC9523609).

### 2.2 APAP model of acute liver injury

In vivo experiments were performed using fasted (approx. 18 h) male C57Bl/6N mice from Charles River Laboratories (Wilmington, MA). Acute liver injury was induced via a single intraperitoneal injection of 300 mg/kg of APAP as described previously (McMillin et al., 2019). A subgroup of mice was injected with 5 mg/kg of KU55933 dissolved in 1:3 DMSO:Saline, or 1:3 DMSO:Saline as vehicle, into the peritoneum 1 h prior to APAP injection. To ensure the mice remained in normothermic conditions post‐APAP injection, they were placed on a heating pad and given free access to water. At multiple timepoints following APAP injection, mice were euthanized, and serum and liver tissue were collected. The livers were snap‐frozen or fixed in 10% buffered formalin (Cat# SF98‐4) from Thermo Scientific (Waltham, MA) and embedded in paraffin. This study was carried out in strict accordance with the recommendations in the Guide for the Care and Use of Laboratory Animals of the National Institutes of Health. The protocol was approved by the University of Texas at Austin (Austin, TX) Institutional Animal Care and Use Committee. All efforts were made to minimize suffering.

### 2.3 Cell culture and treatment

FL83B (Cat# CRL‐2390) mouse hepatocytes were obtained from ATCC (Manassas, VA). Cells were cultured in F‐12K Medium (Cat# 21127030) from ThermoFisher (Waltham, MA) and supplemented with 1% penicillin–streptomycin (Cat# 15070060) and 1% L‐Glutamine (Cat# A2916801) and 10% fetal bovine serum (Cat# 26140‐079) from Gibco (Gaithersburg, MD). Cell treatments included APAP from 1 nM to 100 mM concentrations, KU55933 at 20 nM, and MLN4924 at 5 nM for 24 h. The toxicity of APAP on FL83B was determined using an MTT assay with concentrations from 1 nM to 100 mM. An additional MTT assay was used to assess the combined effects of APAP (5 mM) with 20 nM KU55933.

### 2.4 Immunofluorescence

FL83B cells were treated with 5 mM APAP for 24 h on Lab‐Tek II Chamber Slides (Cat# 155382) from ThermoFisher (Waltham, MA). Cells were fixed using methods previously described (Klattenhoff et al., 2017). Incubation with antibodies specific for ATM, phosphorylated ataxia telangiectasia mutated (pATM), pChk2, and γH2AX was performed overnight at 4°C. The following day, the slides were washed with PBS and incubated with fluorescent tagged secondary antibodies, washed, and mounted with ProLong Gold Antifade mountant with DAPI (Cat# P36935) from ThermoFisher (Waltham, MA). All immunofluorescent images were collected on a Leica SP5 confocal microscope (Wetzlar, Germany) at 40× magnification. Immunofluorescence images were analyzed using ImageJ (US National Institute of Health, Bethesda, Maryland) by assessing the total number of positive cells over the total number of cells present per field.

### 2.5 DNA comet assay

FL83B cells were treated with 5 mM APAP for 24 h on Lab‐Tek II Chamber Slides (Cat# 155382) from ThermoFisher (Waltham, MA). DNA comet assay (Cat# 4250‐050‐K) from Trevigen (Gaithersburg, MD) was performed under neutral conditions to detect the total amount of DSBs present after APAP administration in accordance with the manufacturer's protocol. All images were collected on a Leica SP5 confocal microscope (Wetzlar, Germany) at 63× magnification for the DNA comet assay. DNA comet assay was analyzed using OpenComet extension for ImageJ (US National Institution of Health, Bethesda, Maryland).

### 2.6 Protein extraction

In vivo, liver tissue was homogenized using a Miltenyi Biotec (Bergisch Gladbach, Germany) gentleMACS Dissociator and cell lysis buffer. In vitro, cells were trypsinized using TrypLE (Cat# 12604013) from Gibco (Gaithersburg, MD), washed with PBS, and then lysed using cell lysis buffer. Total protein was quantified using a Pierce BCA Protein Assay kit (Cat# 23227) from ThermoFisher (Waltham, MA) and then stored at −80°C.

### 2.7 Assessment of liver enzymes/metabolites

Serum alanine aminotransferase (ALT) (Cat# 98‐11067‐01) and aspartate aminotransferase (AST) (Cat# 98‐11069‐01) were measured using the IDEXX Catalyst One instrument from IDEXX Laboratories, Inc. (Houston, TX). Total liver GSH levels were measured using a GSH Assay Kit (Cat# ab239709) from Abcam (Cambridge, United Kingdom). The total input for the GSH Assay Kit was 50 mg of liver tissue and was performed according to the manufacturer's instructions, and data was expressed as relative to the control group.

### 2.8 Immunohistochemistry

Paraffin‐embedded livers were prepared and stained following methods previously described (McMillin et al., 2019). Primary antibodies used were for pATM (1:250), pChk2 (1:250), γH2AX (1:500), pSMAD2 (1:100), pSMAD3 (1:100), TGFβ1 (1:100), and p‐c‐cbl (1:100). Slides were then scanned using a Leica Aperio AT2 scanner (Wetzlar, Germany), and images captured at 10x magnification. pATM, pSMAD2, pSMAD3, and TGFβ1 were quantified using ImageJ (National Institute of Health, Bethesda, MD) as a percentage of total area. γH2AX, pChk2, and p‐c‐cbl were calculated by counting the number of positive nuclei over the total number of cells. Threshold values per channel (red, green, and blue) were held consistent between all groups, and those performing the analyses were blind to the groups. For each mouse, 10 field views were quantified and averaged together.

### 2.9 Assessment of liver pathology

Paraffin‐embedded livers were prepared and stained with Hematoxylin and Eosin Y as previously described (McMillin et al., 2019). Slides were then scanned using a Leica Aperio AT2 scanner and images taken at 10× magnification. Quantification of the percentage of necrotic areas was performed using ImageJ. Threshold values per channel (red, green, and blue) were held consistent between all groups, and those performing the analyses were blind to the groups. For each mouse, 10 field views were quantified and averaged together.

### 2.10 Immunoblotting

Protein was extracted from liver tissues and diluted in Laemmli Sample Buffer (Cat# 161‐0747) from BioRad (Hercules, CA). Samples (20 μg of protein) were run on SDS‐PAGE gels (10% v/v) following previously described protocols (McMillin et al., 2019). Antibodies specific for CYP2E1, TGFβRII, SMAD2, SMAD3, pSMAD2, pSMAD3, and GAPDH were used 1:1000 in 5% BSA followed by appropriate Leica secondary antibodies at 1:10,000 in 5% BSA. AIF, JNK, and pJNK antibodies were used 1:500 in 5% BSA with 1:10,000 in 5% BSA Leica secondary antibodies. All imaging was performed on a BioRad ChemiDoc MP Imaging System (Hercules, CA). Data are expressed as fold changes in fluorescent band intensity of target antibody divided by the loading control GAPDH, SMAD2, or SMAD3 relative to the value of the vehicle group. ImageJ software was used to perform band intensity quantification.

### 2.11 Immunoprecipitation

FL83B cells were treated with 5 mM APAP with and without 20 nM KU55933 for 24 h, and protein was extracted. 200 μg of protein per sample were incubated with NEDD8‐specific antibody and pulled down using Magnetic protein A Beads (Cat# 73778) from Cell Signaling (Danvers, MA) per manufacturer's instructions. The resulting precipitants were diluted in Laemmli Sample Buffer (Cat# 161‐0747) from BioRad (Hercules, CA) and electrophoresed on SDS‐PAGE gels (10% v/v). Protein samples incubated with magnetic protein A beads in the absence of NEDD8‐specific antibody were included as a negative control, along with input samples positive for TGFβRII that were included as a positive control. Antibodies specific for TGFβRII were used at a dilution of 1:1000 in 5% BSA, followed by appropriate Leica secondary antibodies at a dilution of 1:10,000 in 5% BSA. All imaging was performed on a BioRad ChemiDoc MP Imaging System (Hercules, CA). Data are expressed as fold changes in fluorescent band intensity of target antibody divided by the loading control, relative to the value of the untreated group. ImageJ software was used to perform band intensity quantification.

### 2.12 ELISA assays

Protein was extracted from liver tissues and diluted with cell lysis buffer. PathScan Phospho‐SMAD3 (Ser423/425) Sandwich ELISA kit (Cat# 12003) and PathScan Phospho‐SMAD2 (Ser465/467) Sandwich ELISA kit (Cat# 7348) were obtained from Cell Signaling (Danvers, MA). TGFβ1 ELISA Kit (Cat# BMS608) was obtained from Invitrogen (Waltham, MA). The total input protein for each sample was 25 μg for the pSMAD3 kit and 50 μg for the pSMAD2 kit and then performed in accordance with the instructions from Cell Signaling. The TGFβ1 kit used an input protein of 10 μg and was performed in accordance with the manufacturer's instructions, except the acid activation step was skipped to ensure that only activated TGFβ1 was measured in our samples. Absorbance was read using a SpectraMax M5 plate reader from Molecular Devices (Sunnyvale, CA). Values were averaged and reported as relative protein levels in comparison to the vehicle group.

### 2.13 Statistical analysis

All data are reported as means ± SEM. Data were analyzed using a student's *t*‐test when only 2 groups were compared; otherwise, when comparing more than two groups, an ANOVA with a Tukey's multiple comparison post hoc test was used. All analysis was completed using GraphPad Software Inc.'s Prism 8 software (La Jolla, CA). *p* < 0.05 was considered significant.

## 3 RESULTS

### 3.1 Acute APAP exposure induces DNA DSBs and ATM signaling in vivo

**FIGURE 1:**
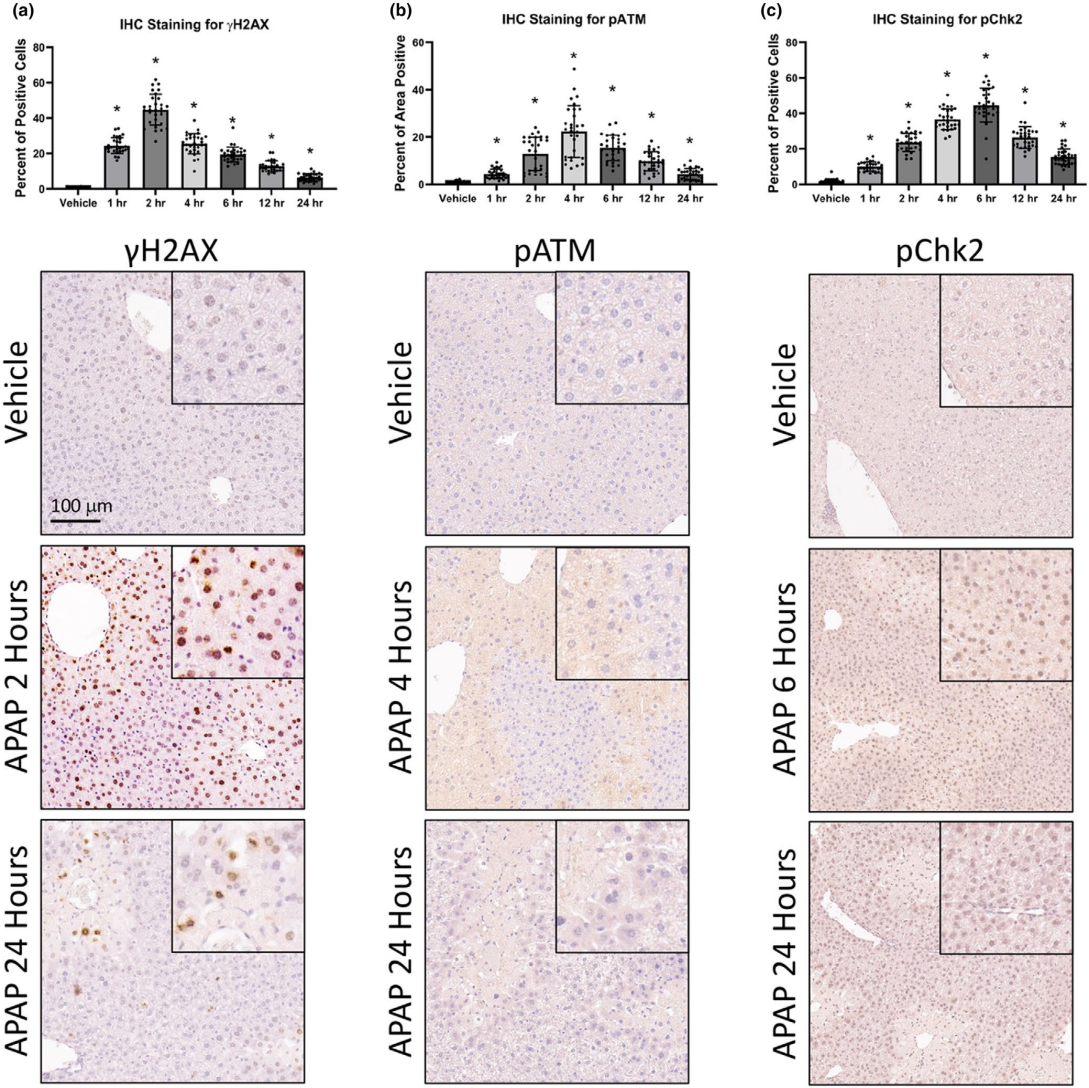
Acute APAP exposure induces DNA double strand breaks and subsequent ATM‐mediated DNA damage response in vivo. (a) Immunohistochemistry quantification and representative images of γH2AX in the liver from mice treated with 300 mg/kg APAP for up to 24 h. (b) Immunohistochemistry quantification and images of pATM in the liver from mice treated with 300 mg/kg APAP for up to 24 h. (c) Immunohistochemistry quantification and images of pChk2 in the liver from mice treated with 300 mg/kg APAP for up to 24 h. Inserted images represent a 20× magnification on a positive area. Data are expressed as average positive staining per field ± SEM (*n* = 10 fields per mouse, from *n* = 3 mice per treatment group) and * denotes *p* values <0.05 compared to vehicle.

Initially experiments were performed to validate that APAP induces hepatic DNA DSBs as has been previously shown (Borude et al., 2018). Mice treated with APAP showed an increase in hepatocellular DNA DSBs. Specifically, there was a significant increase in the number of γH2AX‐positive hepatocytes after APAP exposure with peak immunoreactivity at 2 h post exposure and which remained elevated up to 24 h (Figure 1a). There was a significant increase in pATM expression after APAP injection with peak immunoreactivity occurring at 4 h (Figure 1b). Expression of pChk2 showed a significant increase of immunoreactivity, with a peak occurring at 6‐h post APAP injection, supporting downstream ATM‐mediated DDR signaling is being induced (Figure 1c).

### 3.2 Acute exposure to APAP induces DNA DSBs and ATM signaling in FL83B hepatocytes

To further characterize hepatocyte‐specific consequences of APAP, FL83B cells were used as an in vitro model to investigate ATM‐mediated signaling. FL83B cells were treated with APAP from 1 μM‐100 mM and the LD50 for APAP was found to be approximately 35 mM based off the data from the MTT assay (Figure S1a). A concentration of 5 mM APAP was selected to use for subsequent experiments as this was consistent with previous in vitro studies and was found to induce DNA damage without large amounts of cell death (Pramanick et al., 2021). Further support of this being an effective concentration in these cells was that this APAP concentration significantly increased AIF and pJNK, supporting induction of downstream APAP‐mediated toxicity (Figure S1b,c). This dose of APAP also did not change the expression of CYP2E1 (Figure S1b,c) suggesting that the resulting toxicity was not due alterations in the underlying metabolism of APAP. APAP‐treated FL83B cells showed a significant increase in DNA damage as indicated with increased γH2AX staining with an increase in the number of positive cells from 7.48% to 66.22% (Figure S2a). This was further supported by a neutral DNA comet assay demonstrating evidence of DNA damage (Figure S2b–e). Exposure to 5 mM APAP significantly increased the amount of comet tail DNA (Figure S2c), as well as the comet tail moment (Figure S2d), and the comet olive moment (Figure S2e). The DDR was assessed in FL83B cells and found that pATM and its downstream effector pChk2 were significantly increased in response to 5 mM APAP (Figure S3a,b), indicating an activation of the ATM‐mediated DDR in vitro which is consistent with the in vivo results.

### 3.3 ATM inhibitor KU55933 attenuates the ATM response to APAP in vitro

**FIGURE 2:**
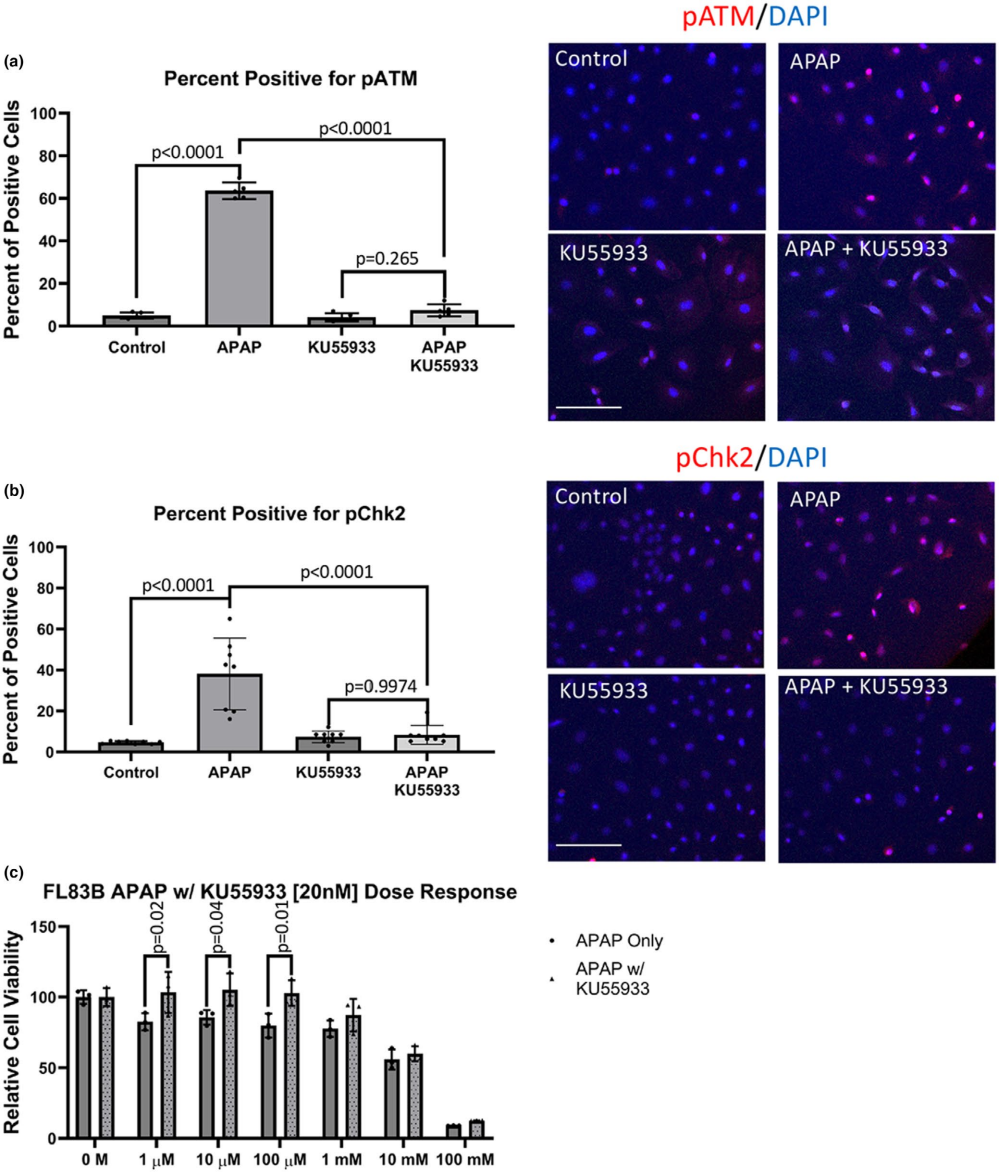
ATM Inhibitor KU55933 attenuates the ATM Response to APAP in vitro. Immunofluorescence images for (a) pATM (red) and (b) pChk2 (red) counterstained with DAPI (blue) at 40× magnification (scale bar is 100 μm). Data are expressed as average number of positive cells per field ± SEM (*n* = 10 fields per group, from *n* = 3 experiments). (c) MTT cell viability assay dose response for FL83B cells to APAP from 1 μM to 100 mM with and without 20 nM KU55933 co‐treatment. Data are expressed as average ± SEM (*n* = 3 replicates per group, from *n* = 3 experiments).

To determine the consequences of pATM‐mediated signaling on APAP toxicity in vitro, the ATM inhibitor KU55933 was utilized. KU55933 did not alter the expression of ATM with or without APAP (data not shown). Treatment with KU55933 significantly attenuated the APAP‐induced increase of pATM immunoreactivity with no significant immunoreactivity observed with cells treated with KU55933 alone (Figure 2a). The inhibition of pATM in cells treated with KU55933 resulted in a significant attenuation of pChk2 expression in response to APAP and no significant difference was observed compared to cells treated with KU55933 only or in combination with APAP (Figure 2b). Additionally, KU55933 significantly attenuated the toxicity of lower dosages of APAP from 1 μM to 100 μM (Figure 2c).

### 3.4 ATM inhibitor KU55933 attenuates the liver damage due to APAP in vivo

**FIGURE 3:**
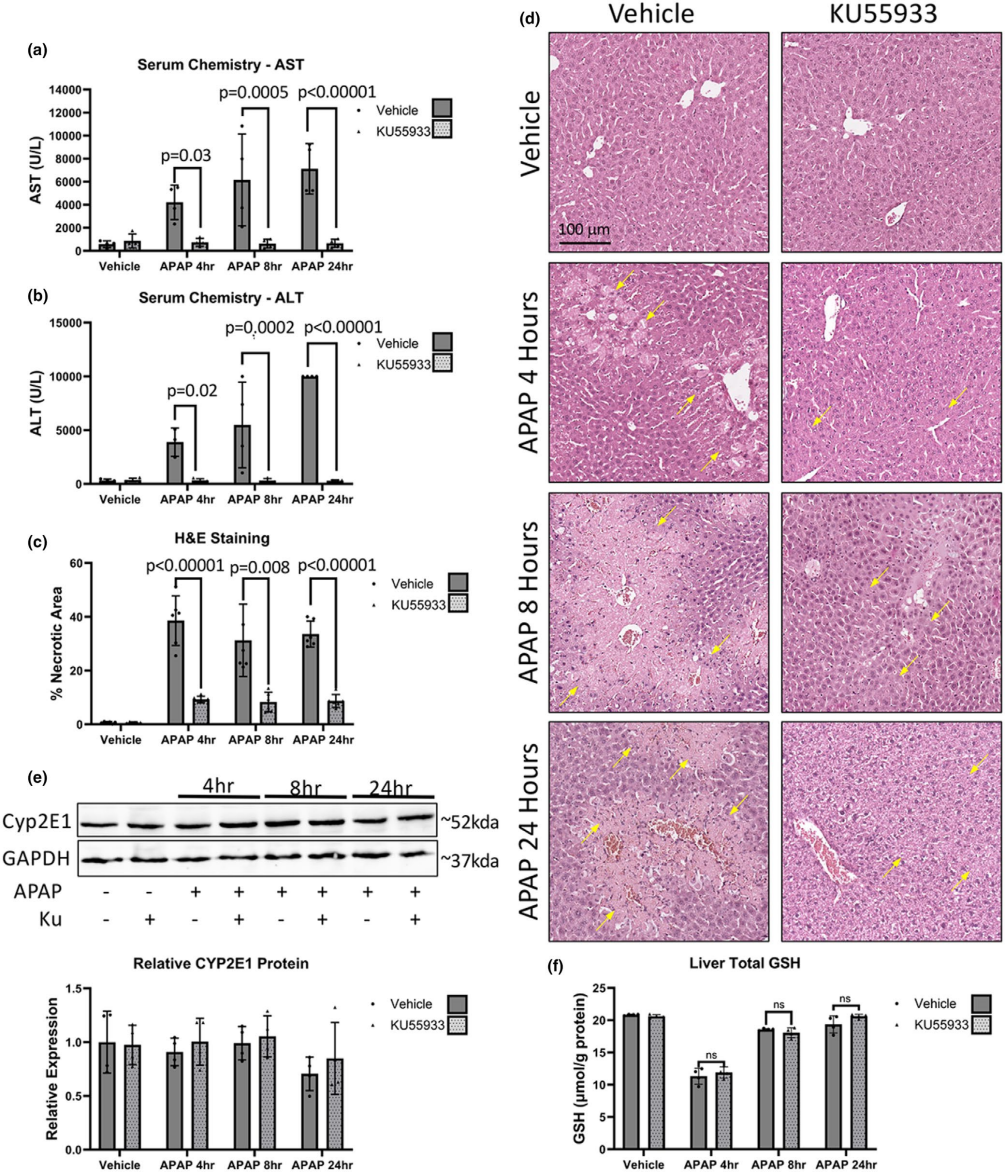
Pretreatment of mice with the KU55933 attenuates liver damage due to APAP in vivo. Mice were treated with 300 mg/kg APAP and a combination with/without a 1‐h pretreatment of 5 mg/kg KU55933. (a) Serum AST and (b) ALT values from mice treated with APAP, KU55933, or appropriate vehicle. (c) Percentage of necrotic liver area was assessed in mice treated with vehicle/APAP and vehicle/KU55933 via H&E with (d) representative images displayed at 10× magnification. Data are expressed as average percent necrotic area per field ± SEM (*n* = 10 fields per mouse, from *n* = 4 mice per treatment group). (e) Representative western blot image and analysis for CYP2E1 in the liver protein lysates from mice treated with APAP, KU55933, or vehicle with GAPDH used as a loading control. (f) Liver GSH levels in mice treated with APAP, KU55933, or vehicle. Data for ALT, AST, CYP2E1, and GSH experiments are expressed as average ± SEM (*n* = 4). n.s, not significant.

To determine if ATM inhibition can also attenuate hepatotoxicity in vivo, mice were pretreated with KU55933 prior to APAP administration to ensure inhibition of ATM phosphorylation. KU55933 pretreatment significantly attenuated the APAP induced increase of ALT and AST at 4 h, at 8 h and at 24 h (Figure 3a,b). H&E staining indicated a significant attenuation of APAP‐induced necrotic area by KU55933 pretreatment at 4 h, 8 h, and at 24 h (Figure 3c,d). To determine if KU55933 is exerting its protective effects via mechanisms that interfere with the metabolism and clearance of APAP, CYP2E1 and GSH levels were evaluated. No significant differences in the relative expression of CYP2E1 were seen between the vehicle and KU only treated groups at all time points studied (Figure 3e). GSH showed no significant difference between groups treated with and without KU55933 at all time points (Figure 3f). Taken together, these data suggest that KU55933 is not interfering with the metabolism or clearance of APAP.

### 3.5 Inhibition of the ATM‐mediated DDR attenuates APAP induced hepatic TGFβ1 signaling in vivo

**FIGURE 4:**
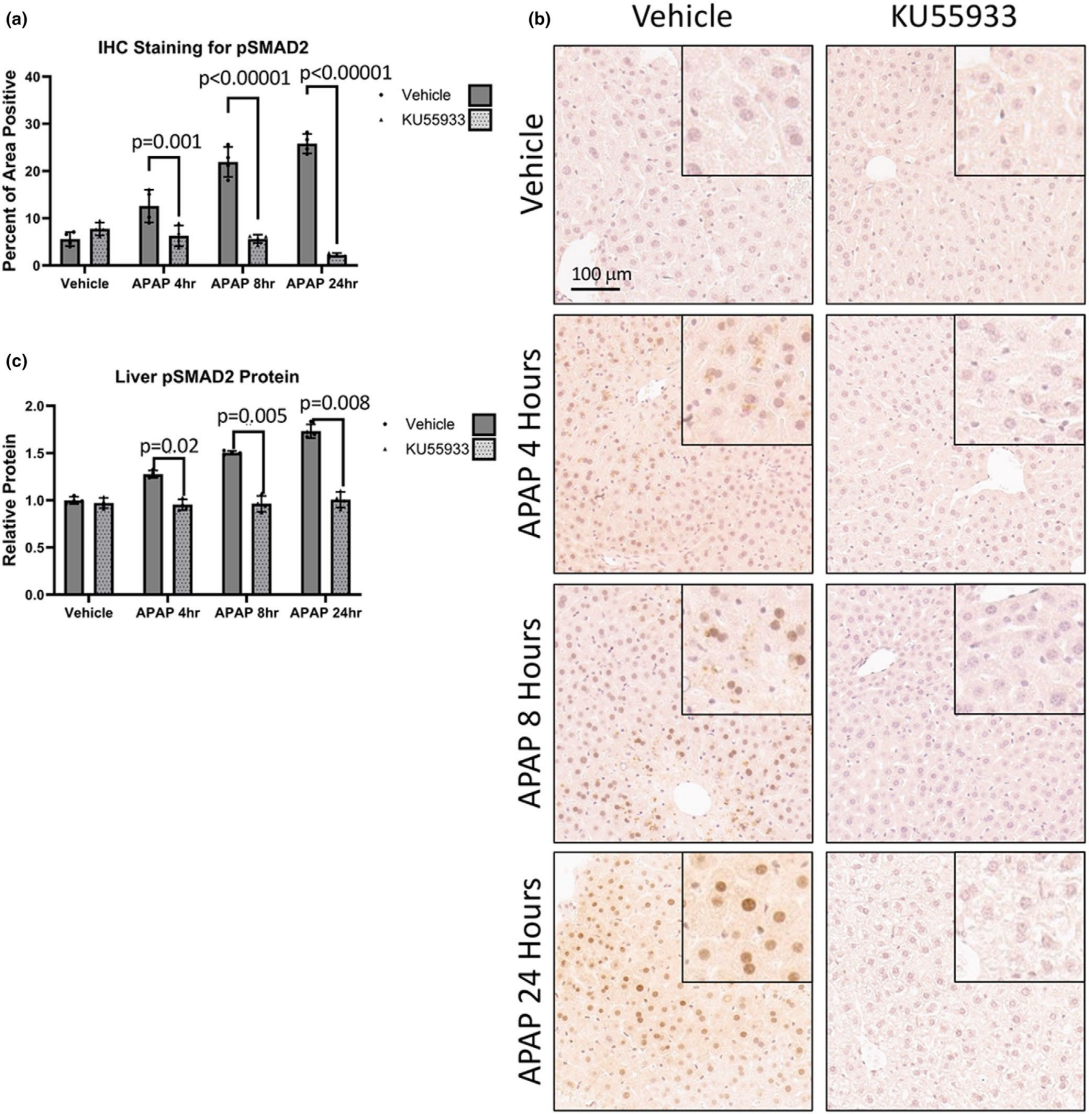
ATM inhibitor KU55933 attenuates APAP‐induced SMAD2 signaling in vivo. (a) Immunohistochemistry quantification of pSMAD2 and (b) representative images of pSMAD2 at 10× magnification from livers of APAP, KU55933, or vehicle‐treated mice. Inserted images represent a 20× magnification on a positive area. Data are expressed as average percent positive area per field ± SEM (*n* = 10 fields per mouse, from *n* = 4 mice per treatment group). (c) ELISA liver pSMAD2 protein levels from APAP, KU55933, or vehicle‐treated mice. Data are expressed as average relative protein levels compared to the control ± SEM (*n* = 4).

**FIGURE 5:**
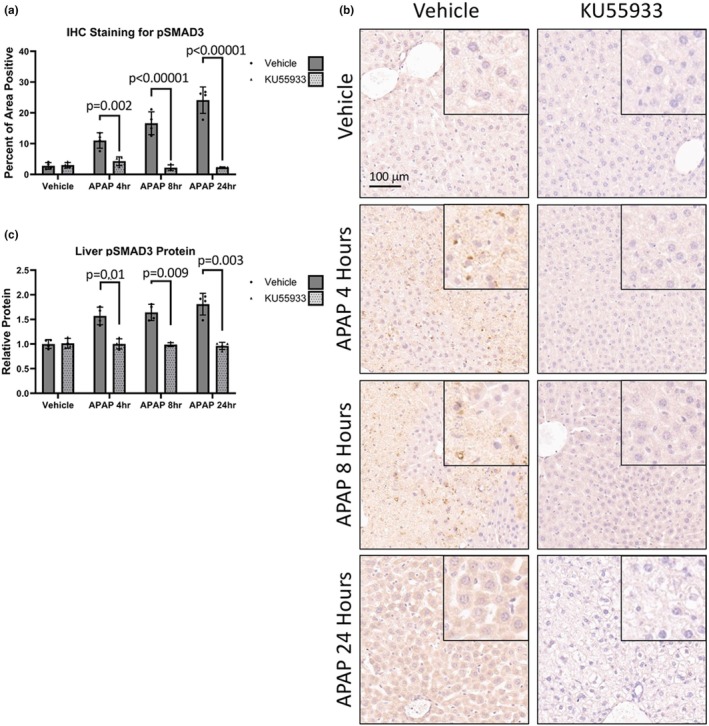
KU55933 attenuates APAP‐induced SMAD3 signaling in vivo. (a) Immunohistochemistry quantification of pSMAD3 and (b) representative images of pSMAD3 at 10× magnification from livers of APAP, KU55933, or vehicle‐treated mice. Inserted images represent a 20× magnification of a positive area. Data are expressed as average percent positive area per field ± SEM (*n* = 10 fields per mouse, from *n* = 4 mice per treatment group). (c) ELISA Liver pSMAD3 protein levels from APAP, KU55933, or vehicle‐treated mice. Data are expressed as average relative protein levels compared to the control ± SEM (*n* = 4).

The ATM‐mediated DDR is involved in regulation of TGFβ1 signaling and this pathway contributes to APAP‐induced toxicity via phosphorylation and activation of SMAD2 and SMAD3 (Li et al., 2019; McMillin et al., 2019). Hepatic pSMAD2 immunostaining showed a significant attenuation after KU55933 administration to APAP‐treated mice at 4 h, 8 h, and at 24 h (Figure 4a,b). The relative amount of hepatic pSMAD2 protein assessed via ELISA had the same trend with a significant attenuation of pSMAD2 at all timepoints studied after KU55933 treatment (Figure 4c). Similarly, pSMAD3 immunoreactivity followed the same pattern with hepatic pSMAD3 immunoreactivity being significantly attenuated by KU55933 administration to APAP‐treated mice at 4 h, at 8 h, and at 24 h (Figure 5a,b). Furthermore, the relative amount of hepatic pSMAD3 protein assessed via ELISA showed a significant attenuation by KU55933 administration at all time points (Figure 5c). These data suggests that ATM‐mediated DDR plays a role in the propagation of TGFβ1 signaling via SMAD2/3 phosphorylation in response to APAP.

### 3.6 ATM inhibitor KU55933 does not alter hepatic TGFβ1 levels in vivo

**FIGURE 6:**
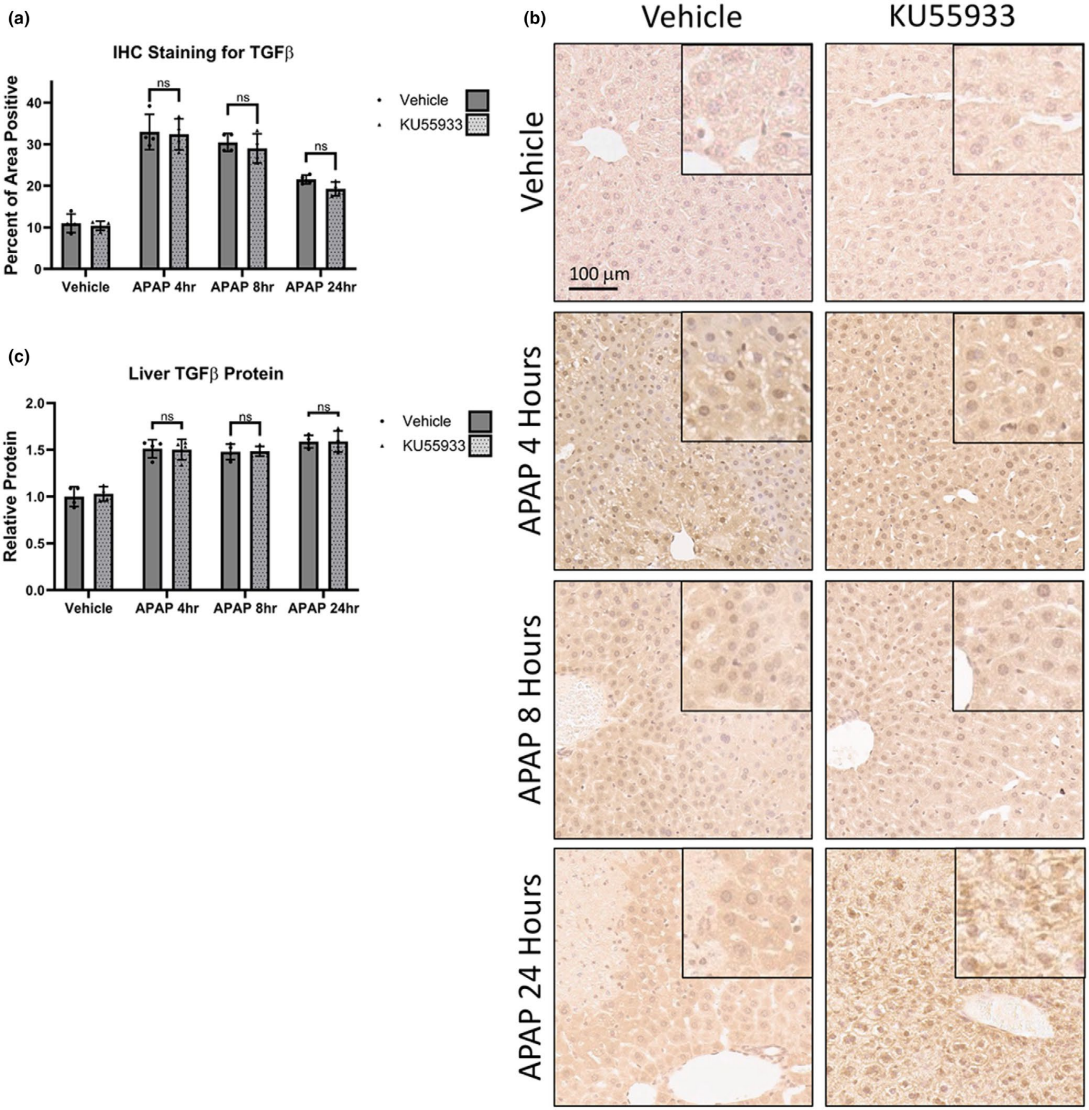
TGFβ1 expression in vivo is unchanged by treatment with KU55933. (a) Immunohistochemistry quantification of TGFβ1 from APAP, KU55933, or vehicle‐treated mice and (b) representative images at 10× magnification with inserted images representing a 20× magnification on a positive area. Data are expressed as average percent positive area per field ± SEM (*n* = 10 fields per mouse, from *n* = 4 mice per treatment group). (c) Activated liver TGFβ1 protein levels from APAP, KU55933, or vehicle‐treated mice assessed via ELISA. Data are expressed as average relative protein levels compared to the control ± SEM (*n* = 4); n.s., not significant.

Hepatic TGFβ1 protein expression was assessed to determine if the associated changes in pSMAD2/3 signaling observed with ATM inhibition were a result of direct alterations in hepatic TGFβ1. TGFβ1 immunoreactivity was increased after APAP injection, however pretreatment with KU55933 had no significant effect on the APAP‐induced increase of TGFβ1 at all time points (Figure 6a,b). To further confirm these results, hepatic protein extracts were assessed for active TGFβ1 via ELISA. Once again, there was no significant difference in the relative amount of active TGFβ1 after APAP between mice treated with and without KU55933 (Figure 6c). This indicates that the attenuation of pSMAD2/3 signaling is not a result of differences in hepatic TGFβ1 production.

### 3.7 APAP induces TGFβ1 signaling via ATM‐mediated neddylation of TGFβRII

**FIGURE 7:**
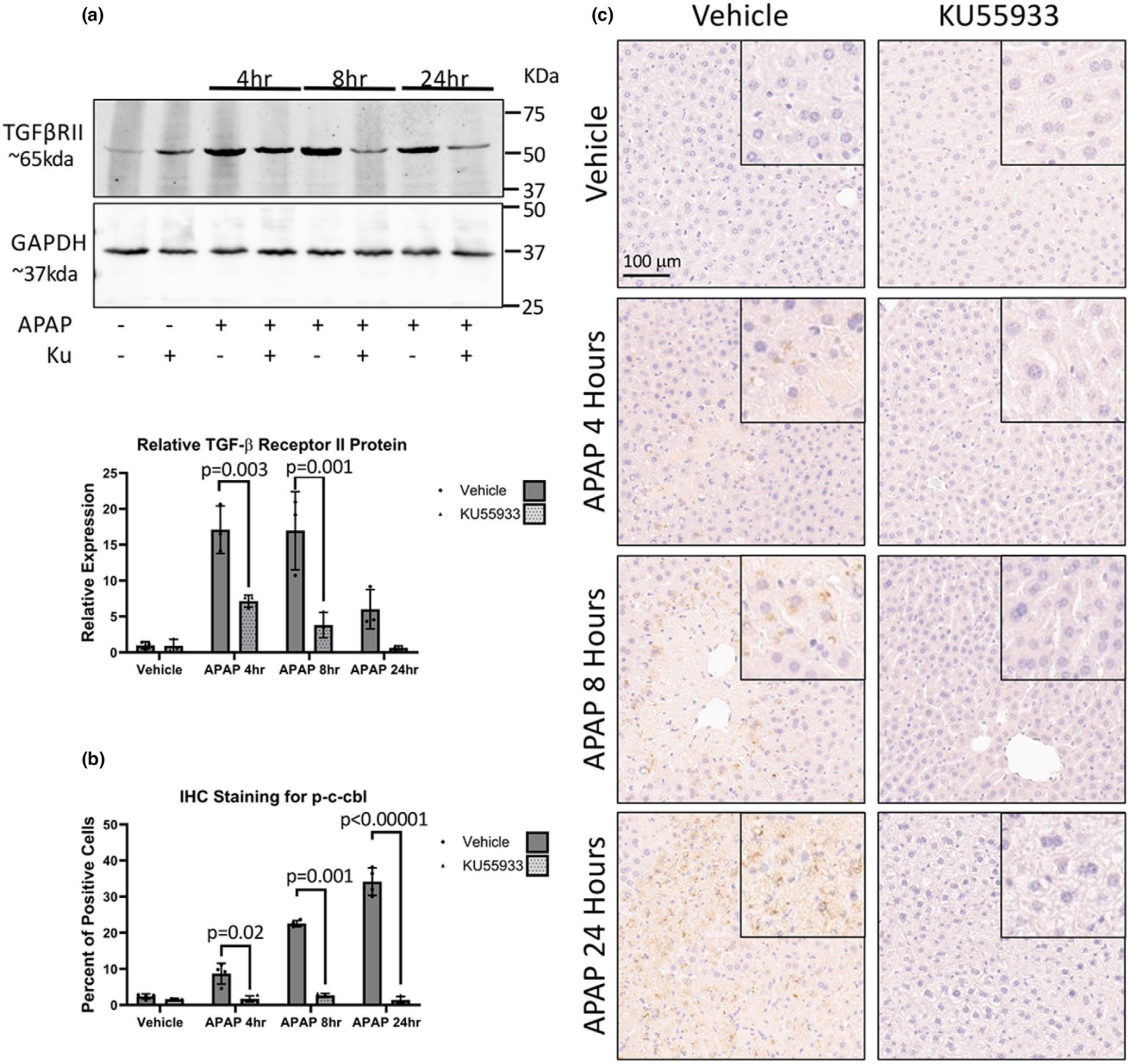
Antagonism of ATM signaling reduces TGFβRII stabilization. (a) Western blot images for TGFβRII in liver protein lysates from APAP, KU55933, or vehicle‐treated mice with GAPDH used as a loading control. (b) Immunohistochemistry quantification of p‐c‐cbl from APAP, KU55933, or vehicle‐treated mice and (c) representative images at 10× magnification with inserted images representing a 20× magnification on a positive area. Data are expressed as average percent positive area per field ± SEM (*n* = 10 fields per mouse, from *n* = 4 mice per treatment group).

DNA damage induced‐ATM signaling can promote stabilization of TGFβRII through c‐cbl phosphorylation and the subsequent neddylation of TGFβRII (Li et al., 2019). To further elucidate the mechanism by which ATM regulates TGFβ1 signaling, ATM‐mediated TGFβRII stabilization was assessed. APAP induces an increase in TGFβRII protein expression at 4, 8, and 24 h, which was attenuated by ATM inhibition (Figure 7a). Immunoreactivity of p‐c‐cbl demonstrated a significant increase in hepatocytes positive for p‐c‐cbl after APAP at all time points studied peaking at 24 h (Figure 7b,c). Inhibition of ATM attenuated the increase in p‐c‐cbl immunoreactivity observed with APAP with no significant difference between KU55933 co‐treatment and vehicle at all time points.

**FIGURE 8:**
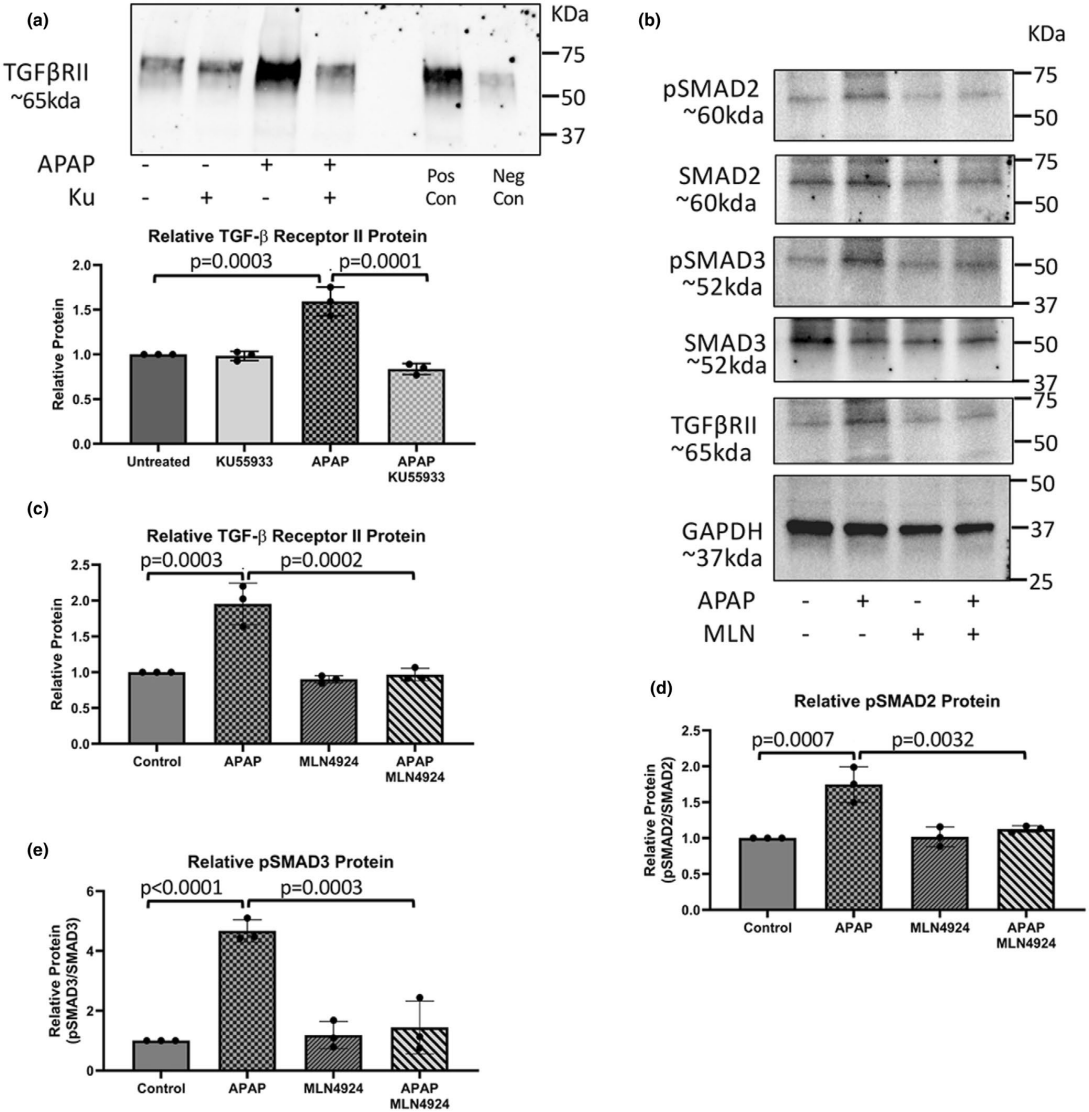
Neddylation of TGFβRII is induced by APAP in vitro. (a) Protein lysates from FL83B cells treated with 5 mM APAP in combination with/without 20 nM KU55933 underwent immunoprecipitation using a NEDD8‐specific antibody followed by immunoblotting for TGFβRII. Data are expressed as average neddylated TGFβRII relative to the control. (b) Representative immunoblot images for pSMAD2, SMAD2, pSMAD3, SMAD3, TGFβRII, and GAPDH in FL83B cells treated with 5 mM APAP with or without 5 nM MLN4924. (c) Immunoblot quantification for TGFβRII normalized to GAPDH, (d) pSMAD2 normalized to SMAD2, and (e) pSMAD3 normalized to SMAD3 in FL83B cells treated with 5 mM APAP with or without 5 nM MLN4924. Data are expressed as relative protein expression ± SEM (*n* = 4).

To determine the neddylation of TGFβRII, FL83B cells were treated with APAP in the presence and absence of KU55933 and immunoprecipitation for NEDD8 was performed. There was a significant increase in neddylated TGFβRII with APAP treatment that was attenuated with the inhibition of ATM (Figure 8a). APAP‐treated FL83B cells were treated with MLN4924, a neddylation inhibitor, and demonstrated that the APAP‐induced significant increase of TGFβRII, pSMAD2, and pSMAD3 expression were all attenuated (Figure 8b–e). These data suggest that APAP‐induced ATM signaling induces TGFβ1 downstream signaling via the neddylation and subsequent stabilization of TGFβRII.

## 4 DISCUSSION

The data presented here demonstrates the role ATM activation has on TGFβ1 signaling in APAP‐induced hepatotoxicity. Specifically, we demonstrated that (i) APAP‐induced hepatotoxicity leads to an activation of ATM in both in vitro and in vivo models of acute liver injury, (ii) the use of the small molecule KU55933 inhibits the ATM‐mediated response to APAP‐induced hepatotoxicity, and (iii) ATM plays a crucial role in TGFβ1 signaling through the stabilization of TGFβRII. Taken together, these data indicate that targeting the ATM‐mediated DDR may be a viable option for the development of novel treatment regimens for managing APAP‐induced liver injury.

DNA damage is a detrimental process for the cells to overcome and as a result, cells have developed at least five major repair pathways to alleviate this pathological state (Chatterjee & Walker, 2017). Specifically, ATM‐mediated repair pathways play a crucial role in the repair of DNA DSBs (Chatterjee & Walker, 2017). The data presented in this report indicate that ATM signaling plays a crucial response in APAP‐induced liver injury, DNA damage, and the DDR. The data supports that APAP can elicit DNA DSBs and a subsequent ATM/Chk2 mediated DDR which is consistent with past studies using human carcinomas cells (Bandi et al., 2014; Gupta et al., 2020; Viswanathan et al., 2018, 2021). Furthermore previous studies have shown that a dosage of 300 mg/kg of APAP induces DSBs within the liver (Borude et al., 2018). This accumulation of DSBs from APAP is able to undergo repair and regeneration of the liver. Here we confirm these findings and show that the ATM/Chk2 mediated response is activated within the livers of the mice after APAP, which is consistent with past in vitro studies (Bandi et al., 2014; Gupta et al., 2020; Viswanathan et al., 2018, 2021). This suggests that the ATM/Chk2 mediated DDR plays a direct role in the repair of the DNA damage caused by APAP‐induced hepatotoxicity.

The ATM/Chk2 signaling pathway that was activated during the DDR may lead to cell cycle arrest, apoptosis, and cellular senescence in various cell types (Pitolli et al., 2019; Roos & Kaina, 2006). This ATM signaling pathway has been shown to play a role in other liver disease expressing high levels of oxidative stress such as metabolic dysfunction‐associated steatotic liver disease, hepatitis, and cholangitis (Daugherity et al., 2012; Matsuda et al., 2014; Sasaki et al., 2008). Past studies have used the ATM inhibitor KU55933 both in vitro and in vivo to inhibit ATM signaling in cancers (Tian et al., 2015; Uehara et al., 2020). Here data is presented indicating a therapeutic value for KU55933 in the treatment of APAP‐induced liver injury. Our data show that KU55933 inhibits the ATM signaling response to APAP‐induced hepatotoxicity and in doing so attenuates the resulting damage. Further studies are required to evaluate the potential of KU55933 as an adjunct therapy with current standards of care for APAP overdose.

Another consequence of downstream ATM activation is its interplay with TGFβ1 signaling. ATM activation is responsible for the stabilization of the TGFβRII through p‐c‐cbl‐mediated neddylation (Li et al., 2019). This posttranslational modification prevents the ubiquitination and subsequent degradation of the receptor, allowing for enhanced TGFβ signaling to occur (Li et al., 2019). Previous research has shown that TGFβ1 signaling plays a critical role in the progression of APAP‐induced liver injury, and inhibition of this signaling pathway alleviates injury from APAP (McMillin et al., 2019). Furthermore, TGFβ1 has been shown to contribute to APAP‐induced inhibition of hepatocyte regeneration via the acquisition of senescence phenotypes in the liver (Bird et al., 2018). Consistent with a role for TGFβ1 in APAP‐induced liver pathology, the current study presents data demonstrating an increase in TGFβ1 and TGFβ1‐mediated SMAD2/3 signaling in response to APAP‐induced injury. Additionally, we present novel data indicating an increase in TGFβRII protein levels along with the increase of TGFβRII neddylation following APAP‐induced liver injury, which likely contributes to the role of TGFβ1 in APAP‐induced toxicity.

With the inhibition of ATM, the data shows an attenuation of TGFβ1‐signaling response seen with APAP‐induced liver injury while not altering hepatic TGFβ1 levels. Specifically, KU55933 treatment attenuated the APAP‐induced neddylation of TGFβRII and the activation of SMAD2/3 in vitro and in vivo. This suggests that ATM plays a role in driving the TGFβ1‐mediated response found with APAP‐induced hepatotoxicity. In support of this, TGFβ1 has been shown to induce the ATM‐mediated DDR in response to irradiation and ATM‐mediated p53 signaling in other cell types (Cipriano et al., 2011; Lee et al., 2016; Overstreet et al., 2015). This provides evidence that TGFβ1 could shift the downstream effects of ATM signaling from DNA repair to cellular death.

One limitation of the current study is that the in vivo and in vitro treatments were given prior to APAP exposure. This was to ensure that ATM‐mediated signaling could be inhibited throughout the APAP time course to better characterize its effects on hepatic injury and signaling. It is possible that using KU55933 as a pretreatment may be inducing a larger therapeutic benefit than if it was administered after APAP toxicity is induced. Future studies are warranted to determine the appropriate therapeutic window for inhibition of ATM to better align with clinical presentation and management of APAP toxicity. One other limitation is that this study investigated neddylation of TGFβR2 in cell culture but not in vivo. It is likely that neddylation is occurring in APAP‐treated mice, as expression of p‐c‐cbl, which is an upstream driver of neddylation, is increased. Future studies will be needed to objectively investigate mechanisms and regulation of TGFβR2 neddylation in vivo.

In summary, the data presented suggest that during APAP‐induced hepatotoxicity there is an accumulation of DNA damage in the form of DSBs, which in turn leads to an ATM‐mediated DDR and ATM‐mediated TGFβ1 signaling, ultimately resulting in hepatocellular death. The use of an ATM inhibitor alleviated the damage caused from APAP‐induced liver injury and an attenuation of TGFβ1‐mediated SMAD signaling. Strategies to inhibit the DDR or ATM signaling may help in the regenerative response to APAP and may be a viable target for the development for adjunct therapy for the management of APAP overdose.

## AUTHOR CONTRIBUTIONS

Matthew McMillin: Conceptualization, validation, and writing original draft; Christopher S Chu: Conceptualization, investigation, and writing original draft; Elaina Williams, Juliet Venter, Kiersten Bell, Anca Petrescu, and Patrick Mireles: Investigation, writing, reviewing, and editing, Sharon DeMorrow: Conceptualization, validation, supervision, finding acquisition, writing, reviewing, and editing.

## FUNDING INFORMATION

This study was funded by NIH R01 awards (DK112803 and DK135995) to SD. The funders had no role in study design, data collection and analysis, decision to publish, or preparation of the manuscript.

## CONFLICT OF INTEREST STATEMENT

The authors have no conflicts of interest to report.

## ETHICS STATEMENT

All experimental procedures involving animals were conducted in strict adherence to the guidelines set forth by the University of Texas at Austin‘s Animal Care and Use Committee (IACUC), with approval number [2024‐00322]. Efforts were made to minimize animal discomfort, aligning with the principle of respect for life in physiological research.

## Supporting information


Figures S1–S3.

## Data Availability

Data are available on request from the corresponding author.

## References

[phy270695-bib-0001] Abdollah, S. , Macías‐Silva, M. , Tsukazaki, T. , Hayashi, H. , Attisano, L. , & Wrana, J. L. (1997). TbetaRI phosphorylation of Smad2 on Ser465 and Ser467 is required for Smad2‐Smad4 complex formation and signaling. Journal of Biological Chemistry, 272(44), 27678–27685. 9346908 10.1074/jbc.272.44.27678

[phy270695-bib-0002] Bandi, S. , Viswanathan, P. , & Gupta, S. (2014). Evaluation of cytotoxicity and DNA damage response with analysis of intracellular ATM signaling pathways. Assay and Drug Development Technologies, 12(5), 272–281. 24927134 10.1089/adt.2014.571 PMC4060777

[phy270695-bib-0003] Bernal, W. , Auzinger, G. , Dhawan, A. , & Wendon, J. (2010). Acute liver failure. Lancet, 376(9736), 190–201. 20638564 10.1016/S0140-6736(10)60274-7

[phy270695-bib-0004] Bird, T. G. , Müller, M. , Boulter, L. , Vincent, D. F. , Ridgway, R. A. , Lopez‐Guadamillas, E. , Lu, W. Y. , Jamieson, T. , Govaere, O. , Campbell, A. D. , Ferreira‐Gonzalez, S. , Cole, A. M. , Hay, T. , Simpson, K. J. , Clark, W. , Hedley, A. , Clarke, M. , Gentaz, P. , Nixon, C. , … Forbes, S. J. (2018). TGFβ inhibition restores a regenerative response in acute liver injury by suppressing paracrine senescence. Science Translational Medicine, 10(454), eaan1230. 30111642 10.1126/scitranslmed.aan1230 PMC6420144

[phy270695-bib-0005] Borude, P. , Bhushan, B. , & Apte, U. (2018). DNA damage response regulates initiation of liver regeneration following acetaminophen overdose. Gene Expression, 18(2), 115–123. 29540258 10.3727/105221618X15205260749346 PMC5954624

[phy270695-bib-0006] Chatterjee, N. , & Walker, G. C. (2017). Mechanisms of DNA damage, repair, and mutagenesis. Environmental and Molecular Mutagenesis, 58(5), 235–263. 28485537 10.1002/em.22087 PMC5474181

[phy270695-bib-0007] Chou, W. C. , Hu, L. Y. , Hsiung, C. N. , & Shen, C. Y. (2015). Initiation of the ATM‐Chk2 DNA damage response through the base excision repair pathway. Carcinogenesis, 36(8), 832–840. 26025911 10.1093/carcin/bgv079

[phy270695-bib-0008] Chu, C. S. (2023). ATM‐mediated DNA damage response promotes acetaminophen‐induced acute liver injury through mediation of TGFβ signaling. The University of Texas at Austin, Texas ScholarWorks. 10.26153/tsw/59176

[phy270695-bib-0009] Cipriano, R. , Kan, C. E. , Graham, J. , Danielpour, D. , Stampfer, M. , & Jackson, M. W. (2011). TGF‐beta signaling engages an ATM‐CHK2‐p53‐independent RAS‐induced senescence and prevents malignant transformation in human mammary epithelial cells. Proceedings of the National Academy of Sciences of the United States of America, 108(21), 8668–8673. 21555587 10.1073/pnas.1015022108 PMC3102347

[phy270695-bib-0010] Cortez, D. , Guntuku, S. , Qin, J. , & Elledge, S. J. (2001). ATR and ATRIP: partners in checkpoint signaling. Science, 294(5547), 1713–1716. 11721054 10.1126/science.1065521

[phy270695-bib-0011] Dahlin, D. C. , Miwa, G. T. , Lu, A. Y. , & Nelson, S. D. (1984). N‐acetyl‐p‐benzoquinone imine: A cytochrome P‐450‐mediated oxidation product of acetaminophen. Proceedings of the National Academy of Sciences of the United States of America, 81(5), 1327–1331. 6424115 10.1073/pnas.81.5.1327 PMC344826

[phy270695-bib-0012] Daugherity, E. K. , Balmus, G. , Al Saei, A. , Moore, E. S. , Abi Abdallah, D. , Rogers, A. B. , Weiss, R. S. , & Maurer, K. J. (2012). The DNA damage checkpoint protein ATM promotes hepatocellular apoptosis and fibrosis in a mouse model of non‐alcoholic fatty liver disease. Cell Cycle, 11(10), 1918–1928. 22544329 10.4161/cc.20259 PMC3359121

[phy270695-bib-0013] Dizdaroglu, M. (2012). Oxidatively induced DNA damage: mechanisms, repair and disease. Cancer Letters, 327(1–2), 26–47. 22293091 10.1016/j.canlet.2012.01.016

[phy270695-bib-0014] Ghanem, C. I. , Pérez, M. J. , Manautou, J. E. , & Mottino, A. D. (2016). Acetaminophen from liver to brain: New insights into drug pharmacological action and toxicity. Pharmacological Research, 109, 119–131. 26921661 10.1016/j.phrs.2016.02.020 PMC4912877

[phy270695-bib-0015] Gupta, P. , Sharma, Y. , Viswanathan, P. , & Gupta, S. (2020). Cellular cytokine receptor signaling and ATM pathway intersections affect hepatic DNA repair. Cytokine, 127, 154946. 31837586 10.1016/j.cyto.2019.154946

[phy270695-bib-0016] Jollow, D. J. , Mitchell, J. R. , Potter, W. Z. , Davis, D. C. , Gillette, J. R. , & Brodie, B. B. (1973). Acetaminophen‐induced hepatic necrosis. II. Role of covalent binding in vivo. Journal of Pharmacology and Experimental Therapeutics, 187(1), 195–202. 4746327

[phy270695-bib-0017] Kaufman, D. W. , Kelly, J. P. , Rosenberg, L. , Anderson, T. E. , & Mitchell, A. A. (2002). Recent patterns of medication use in the ambulatory adult population of the United States: the Slone survey. JAMA, 287(3), 337–344. 11790213 10.1001/jama.287.3.337

[phy270695-bib-0018] Klattenhoff, A. W. , Thakur, M. , Chu, C. S. , Ray, D. , Habib, S. L. , & Kidane, D. (2017). Loss of NEIL3 DNA glycosylase markedly increases replication associated double strand breaks and enhances sensitivity to ATR inhibitor in glioblastoma cells. Oncotarget, 8(68), 112942–112958. 29348879 10.18632/oncotarget.22896 PMC5762564

[phy270695-bib-0019] Lee, J. , Kim, M. R. , Kim, H. J. , An, Y. S. , & Yi, J. Y. (2016). TGF‐β1 accelerates the DNA damage response in epithelial cells via Smad signaling. Biochemical and Biophysical Research Communications, 476(4), 420–425. 27237972 10.1016/j.bbrc.2016.05.136

[phy270695-bib-0020] Lee, W. M. (2013). Drug‐induced acute liver failure. Clinics in Liver Disease, 17(4), 575–586. 24099019 10.1016/j.cld.2013.07.001 PMC3838908

[phy270695-bib-0021] Li, Y. , Liu, Y. , Chiang, Y. J. , Huang, F. , Li, Y. , Li, X. , Ning, Y. , Zhang, W. , Deng, H. , & Chen, Y. G. (2019). DNA damage activates TGF‐β signaling via ATM‐c‐Cbl‐mediated stabilization of the Type II receptor TβRII. Cell Reports, 28(3), 735–745. 31315051 10.1016/j.celrep.2019.06.045

[phy270695-bib-0022] Manyike, P. T. , Kharasch, E. D. , Kalhorn, T. F. , & Slattery, J. T. (2000). Contribution of CYP2E1 and CYP3A to acetaminophen reactive metabolite formation. Clinical Pharmacology and Therapeutics, 67(3), 275–282. 10741631 10.1067/mcp.2000.104736

[phy270695-bib-0023] Matsuda, Y. , Sanpei, A. , Wakai, T. , Kubota, M. , Osawa, M. , Hirose, Y. , Sakata, J. , Kobayashi, T. , Fujimaki, S. , Takamura, M. , Yamagiwa, S. , Yano, M. , Ohkoshi, S. , & Aoyagi, Y. (2014). Hepatitis B virus X stimulates redox signaling through activation of ataxia telangiectasia mutated kinase. International Journal of Clinical and Experimental Pathology, 7(5), 2032–2043. 24966912 PMC4069949

[phy270695-bib-0024] Matsuoka, S. , Ballif, B. A. , Smogorzewska, A. , McDonald, E. R., III , Hurov, K. E. , Luo, J. , Bakalarski, C. E. , Zhao, Z. , Solimini, N. , Lerenthal, Y. , & Shiloh, Y. (2007). ATM and ATR substrate analysis reveals extensive protein networks responsive to DNA damage. Science, 316(5828), 1160–1166. 17525332 10.1126/science.1140321

[phy270695-bib-0025] McGill, M. R. , Sharpe, M. R. , Williams, C. D. , Taha, M. , Curry, S. C. , & Jaeschke, H. (2012). The mechanism underlying acetaminophen‐induced hepatotoxicity in humans and mice involves mitochondrial damage and nuclear DNA fragmentation. Journal of Clinical Investigation, 122(4), 1574–1583. 22378043 10.1172/JCI59755 PMC3314460

[phy270695-bib-0026] McMillin, M. , Galindo, C. , Pae, H. Y. , Frampton, G. , Di Patre, P. L. , Quinn, M. , Whittington, E. , & DeMorrow, S. (2014). Gli1 activation and protection against hepatic encephalopathy is suppressed by circulating transforming growth factor β1 in mice. Journal of Hepatology, 61(6), 1260–1266. 25046848 10.1016/j.jhep.2014.07.015 PMC4253574

[phy270695-bib-0027] McMillin, M. , Grant, S. , Frampton, G. , Petrescu, A. D. , Williams, E. , Jefferson, B. , & DeMorrow, S. (2019). The TGFβ1 receptor antagonist GW788388 reduces JNK activation and protects against acetaminophen hepatotoxicity in mice. Toxicological Sciences, 170(2), 549–561. 31132129 10.1093/toxsci/kfz122 PMC6821297

[phy270695-bib-0028] Miwa, Y. , Harrison, P. M. , Farzaneh, F. , Langley, P. G. , Williams, R. , & Hughes, R. D. (1997). Plasma levels and hepatic mRNA expression of transforming growth factor‐beta1 in patients with fulminant hepatic failure. Journal of Hepatology, 27(5), 780–788. 9382963 10.1016/s0168-8278(97)80313-3

[phy270695-bib-0029] Overstreet, J. M. , Samarakoon, R. , Cardona‐Grau, D. , Goldschmeding, R. , & Higgins, P. J. (2015). Tumor suppressor ataxia telangiectasia mutated functions downstream of TGF‐β1 in orchestrating profibrotic responses. The FASEB Journal, 29(4), 1258–1268. 25480384 10.1096/fj.14-262527 PMC4396616

[phy270695-bib-0030] Pitolli, C. , Wang, Y. , Candi, E. , Shi, Y. , Melino, G. , & Amelio, I. (2019). p53‐mediated tumor suppression: DNA‐damage response and alternative mechanisms. Cancers, 11(12), 1983. 31835405 10.3390/cancers11121983 PMC6966539

[phy270695-bib-0031] Potter, D. W. , & Hinson, J. A. (1987). Mechanisms of acetaminophen oxidation to N‐acetyl‐P‐benzoquinone imine by horseradish peroxidase and cytochrome P‐450. The Journal of Biological Chemistry, 262(3), 966–973. 3805031

[phy270695-bib-0032] Pramanick, A. , Chakraborti, S. , Mahata, T. , Basak, M. , Das, K. , Verma, S. K. , Sengar, A. S. , Singh, P. K. , Kumar, P. , Bhattacharya, B. , Biswas, S. , Pal, P. B. , Sarkar, S. , Agrawal, V. , Saha, S. , Nath, D. , Chatterjee, S. , Stewart, A. , & Maity, B. (2021). G protein β5‐ATM complexes drive acetaminophen‐induced hepatotoxicity. Redox Biology, 43, 101965. 33933881 10.1016/j.redox.2021.101965 PMC8105674

[phy270695-bib-0033] Rasmussen, R. E. , & Painter, R. B. (1964). Evidence for repair of ultra‐violet damaged deoxyribonucleic acid in cultured mammalian cells. Nature, 203, 1360–1362. 14207310 10.1038/2031360a0

[phy270695-bib-0034] Rogakou, E. P. , Pilch, D. R. , Orr, A. H. , Ivanova, V. S. , & Bonner, W. M. (1998). DNA double‐stranded breaks induce histone H2AX phosphorylation on serine 139. The Journal of Biological Chemistry, 273(10), 5858–5868. 9488723 10.1074/jbc.273.10.5858

[phy270695-bib-0035] Roos, W. P. , & Kaina, B. (2006). DNA damage‐induced cell death by apoptosis. Trends in Molecular Medicine, 12(9), 440–450. 16899408 10.1016/j.molmed.2006.07.007

[phy270695-bib-0036] Sasaki, M. , Ikeda, H. , & Nakanuma, Y. (2008). Activation of ATM signaling pathway is involved in oxidative stress‐induced expression of mito‐inhibitory p21WAF1/Cip1 in chronic non‐suppurative destructive cholangitis in primary biliary cirrhosis: an immunohistochemical study. Journal of Autoimmunity, 31(1), 73–78. 18456456 10.1016/j.jaut.2008.03.005

[phy270695-bib-0037] Shen, W. , Kamendulis, L. M. , Ray, S. D. , & Corcoran, G. B. (1992). Acetaminophen‐induced cytotoxicity in cultured mouse hepatocytes: Effects of Ca(2+)‐endonuclease, DNA repair, and glutathione depletion inhibitors on DNA fragmentation and cell death. Toxicology and Applied Pharmacology, 112(1), 32–40. 1310169 10.1016/0041-008x(92)90276-x

[phy270695-bib-0038] Shiloh, Y. , & Ziv, Y. (2013). The ATM protein kinase: Regulating the cellular response to genotoxic stress, and more. Nature Reviews Molecular Cell Biology, 14(4), 197–210. 23847781

[phy270695-bib-0039] Stracker, T. H. , Roig, I. , Knobel, P. A. , & Marjanović, M. (2013). The ATM signaling network in development and disease. Frontiers in Genetics, 4, 37. 23532176 10.3389/fgene.2013.00037 PMC3607076

[phy270695-bib-0040] Sun, Y. , Jiang, X. , Chen, S. , Fernandes, N. , & Price, B. D. (2005). A role for the Tip60 histone acetyltransferase in the acetylation and activation of ATM. Proceedings of the National Academy of Sciences of the United States of America, 102(37), 13182–13187. 16141325 10.1073/pnas.0504211102 PMC1197271

[phy270695-bib-0041] Tian, X. , Lara, H. , Wagner, K. T. , Saripalli, S. , Hyder, S. N. , Foote, M. , Sethi, M. , Wang, E. , Caster, J. M. , Zhang, L. , & Wang, A. Z. (2015). Improving DNA double‐strand repair inhibitor KU55933 therapeutic index in cancer radiotherapy using nanoparticle drug delivery. Nanoscale, 7(47), 20211–20219. 26575637 10.1039/c5nr05869d PMC4664156

[phy270695-bib-0042] Uehara, M. , Kusaba, T. , Ida, T. , Nakai, K. , Nakata, T. , Tomita, A. , Watanabe‐Uehara, N. , Ikeda, K. , Kitani, T. , Yamashita, N. , Kirita, Y. , Matoba, S. , Humphreys, B. D. , & Tamagaki, K. (2020). Pharmacological inhibition of ataxia‐telangiectasia mutated exacerbates acute kidney injury by activating p53 signaling in mice. Scientific Reports, 10(1), 4441. 32157166 10.1038/s41598-020-61456-7 PMC7064514

[phy270695-bib-0043] Viswanathan, P. , Sharma, Y. , Gupta, P. , & Gupta, S. (2018). Replicative stress and alterations in cell cycle checkpoint controls following acetaminophen hepatotoxicity restrict liver regeneration. Cell Proliferation, 51(3), e12445. 29504225 10.1111/cpr.12445 PMC6500460

[phy270695-bib-0044] Viswanathan, P. , Sharma, Y. , Jaber, F. L. , Tchaikovskaya, T. , & Gupta, S. (2021). Transplanted hepatocytes rescue mice in acetaminophen‐induced acute liver failure through paracrine signals for hepatic ATM and STAT3 pathways. The FASEB Journal, 35(4), e21471. 33683737 10.1096/fj.202002421R

[phy270695-bib-0045] Wang, M. , Saha, J. , Hada, M. , Anderson, J. A. , Pluth, J. M. , & O'Neill, P. (2013). Cucinotta FA. Novel Smad proteins localize to IR‐induced double‐strand breaks: Interplay between TGFβ and ATM pathways. Nucleic Acids Research, 41(2), 933–942. 23221633 10.1093/nar/gks1038 PMC3553971

[phy270695-bib-0046] Wiegman, E. M. , Blaese, M. A. , Loeffler, H. , Coppes, R. P. , & Rodemann, H. P. (2007). TGFbeta‐1 dependent fast stimulation of ATM and p53 phosphorylation following exposure to ionizing radiation does not involve TGFbeta‐receptor I signalling. Radiotherapy and Oncology, 83(3), 289–295. 17560675 10.1016/j.radonc.2007.05.013

[phy270695-bib-0047] Zhang, Y. , Feng, X. , We, R. , & Derynck, R. (1996). Receptor‐associated Mad homologues synergize as effectors of the TGF‐beta response. Nature, 383(6596), 168–172. 8774881 10.1038/383168a0

