# Supplementary material for: Ataxia‐telangiectasia mutated activation mediates transforming growth factor beta signaling in acetaminophen‐induced liver injury in mice
Source: Physiol Rep. 2025 Dec 9;13(23):e70695. doi: 10.14814/phy2.70695 (PMC12689461; doi:10.14814/phy2.70695)
Supplement: Supplementary file 1 — Figures S1–S3. [file PHY2-13-e70695-s001.zip › PHYSREP-2025-07-663-T-s01.docx]

**Supplemental Figure Legends**

**Supplemental Figure 1:** FL83B cells induce cell toxicity signaling pathways in response to APAP. (A) MTT cell viability assay to determine the dose response of FL83B cells to APAP from 1 μM to 100 mM. (B) Representative immunoblots for AIF, JNK, pJNK, CYP2E1, and GAPDH in FL83B cells treated with 5 mM APAP for 24 hours. (C) Quantification of relative immunoblot protein for AIF, pJNK, and CYP2E1 normalized to GAPDH. * denotes p values <0.05 compared to vehicle.

**Supplemental Figure 2:** APAP-induced DNA double strand breaks occur in FL83B cells. (A) Immunofluorescence for γH2AX (green) countered stained with DAPI (blue) at 40x magnification, scale is 100 μm. Inserted boxes represent a 4x digital magnification on a positive area. Quantification data are expressed as average number of positive cells per field ± SEM (n=10 fields per group, from n=3 experiments). (B) Representative images of a neutral condition DNA comet assay. (C) Quantification of the amount of tail DNA, (D) the tail moment, and (E) the olive moment. Data for tail DNA, tail moment, and olive moment are expressed as average ± SEM (n=100 fields per treatment group) and * denotes p values <0.05 compared to control.

**Supplemental Figure 3:** ATM-mediated signaling is increased in FL83B cells in response to APAP toxicity. (A) pATM (Red), and (B) pChk2 (red) counterstained with DAPI (blue) at 40x magnification (scale bar is 100 μm). Inserted boxes represent a 4x digital magnification on a positive area. Data are expressed as average number of positive cells per field ± SEM (n=10 fields per group, from n=3 experiments) and * denotes p values <0.05 compared to control.
