# Supplementary figures and images for: Ataxia‐telangiectasia mutated activation mediates transforming growth factor beta signaling in acetaminophen‐induced liver injury in mice
Source: Physiol Rep. 2025 Dec 9;13(23):e70695. doi: 10.14814/phy2.70695 (PMC12689461; doi:10.14814/phy2.70695)

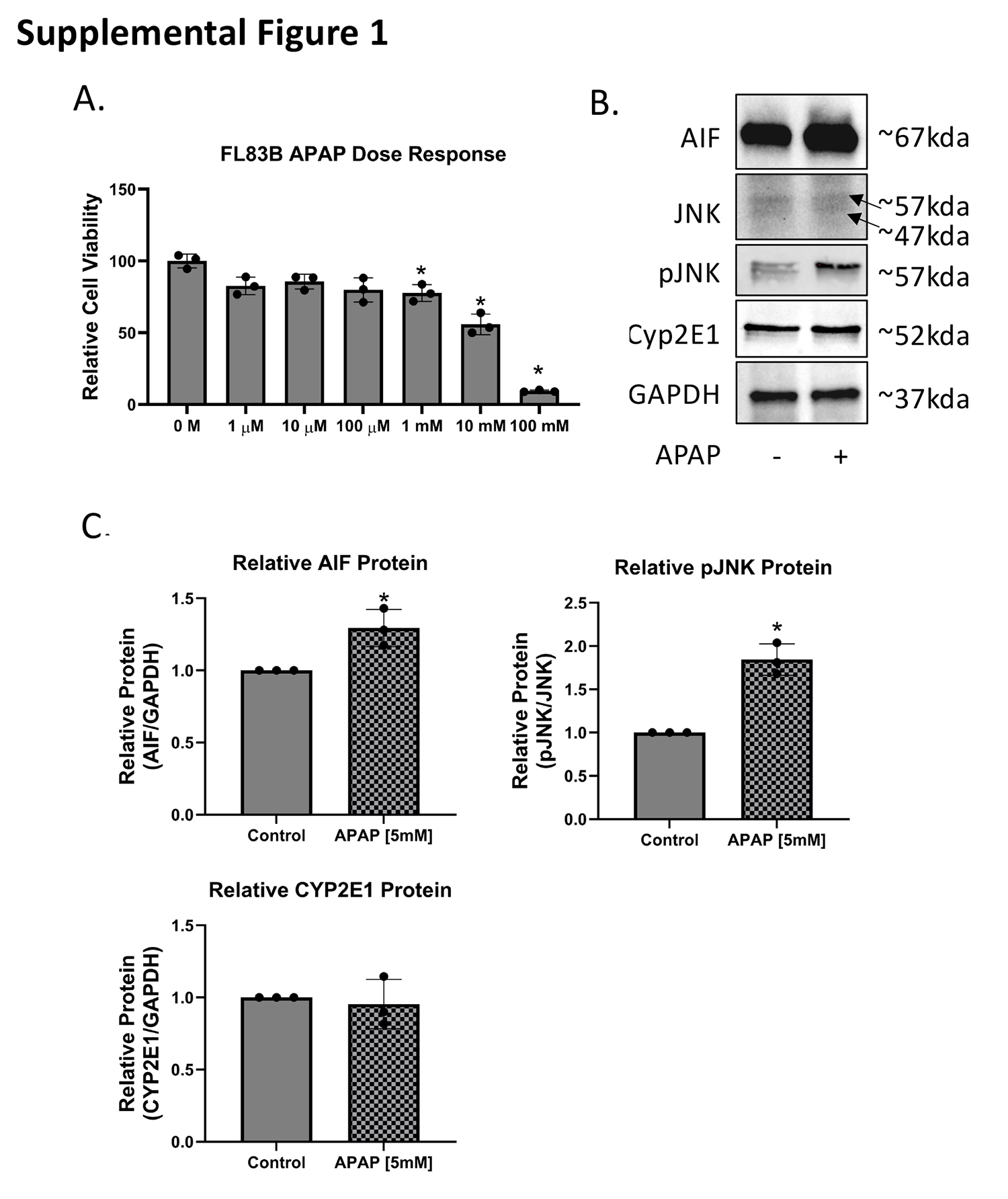

Supplement: Supplementary file 1 — Figures S1–S3. [file PHY2-13-e70695-s001.zip › PHYSREP-2025-07-663-T-s02.tif]

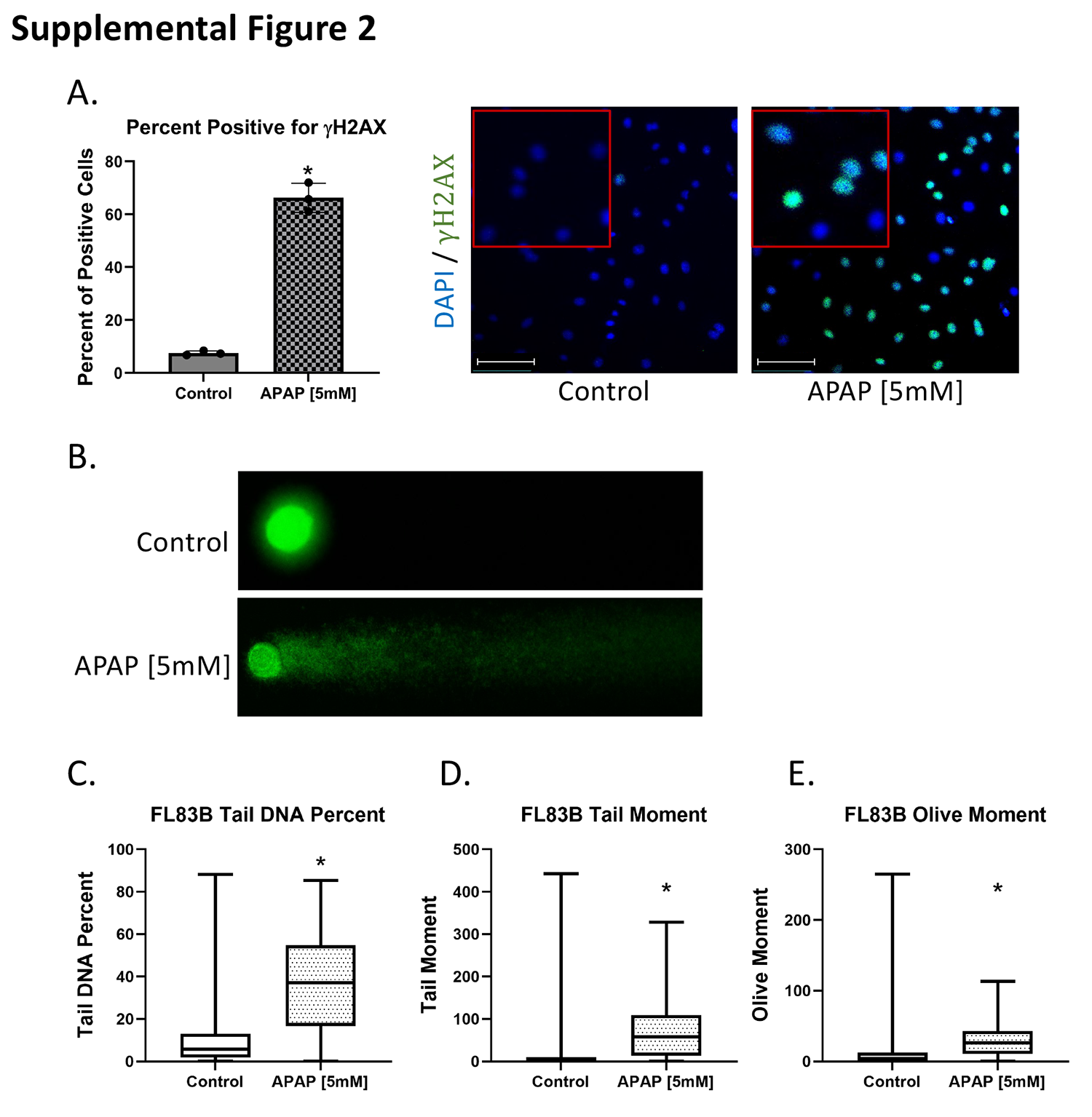

Supplement: Supplementary file 1 — Figures S1–S3. [file PHY2-13-e70695-s001.zip › PHYSREP-2025-07-663-T-s03.tif]

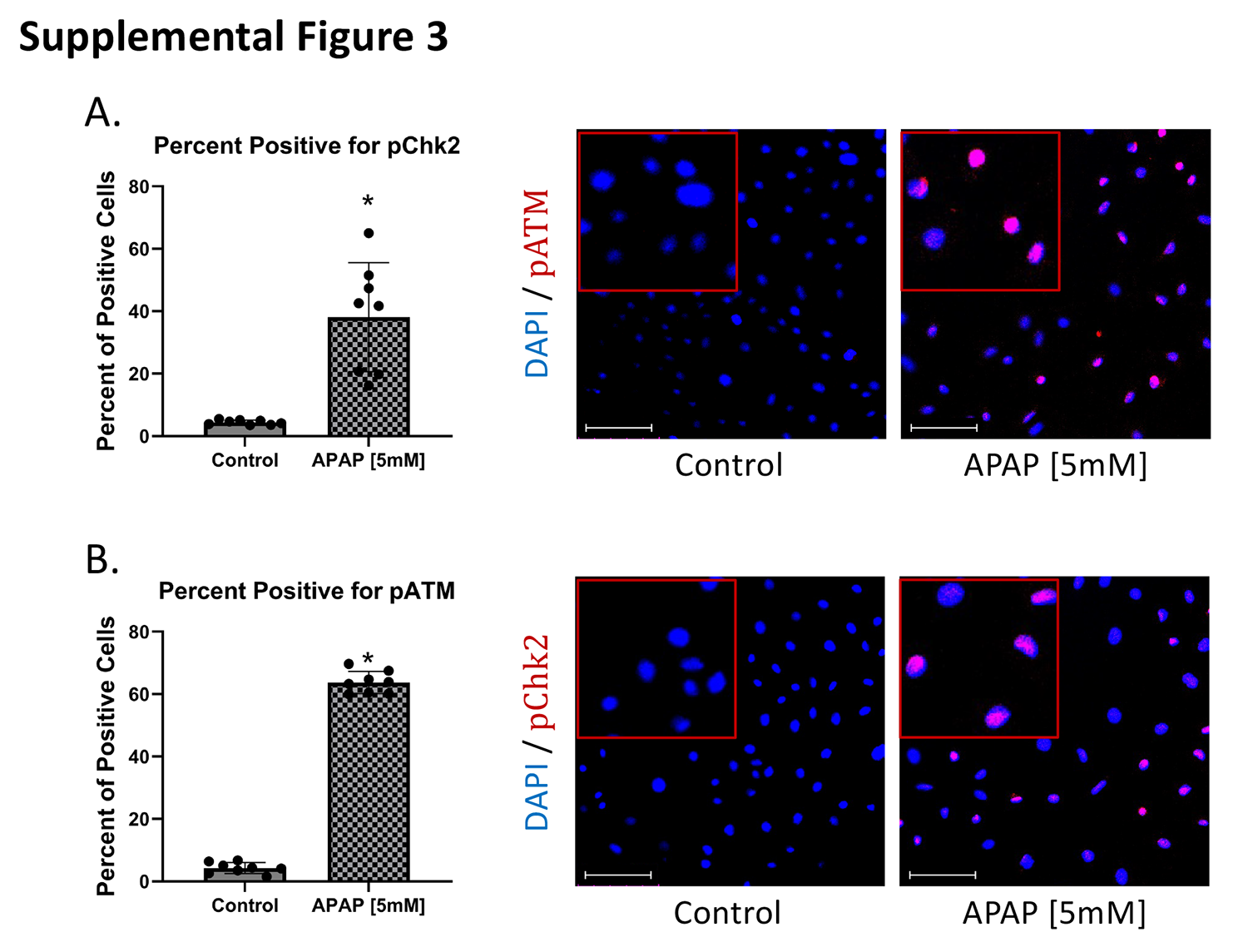

Supplement: Supplementary file 1 — Figures S1–S3. [file PHY2-13-e70695-s001.zip › PHYSREP-2025-07-663-T-s04.tif]
